# Differences in estimates of size distribution of beryllium powder materials using phase contrast microscopy, scanning electron microscopy, and liquid suspension counter techniques

**DOI:** 10.1186/1743-8977-4-3

**Published:** 2007-02-28

**Authors:** Aleksandr B Stefaniak, Mark D Hoover, Robert M Dickerson, Gregory A Day, Patrick N Breysse, Ronald C Scripsick

**Affiliations:** 1 National Institute for Occupational Safety and Health, Division of Respiratory Disease Studies, Mailstop H-2703, 1095 Willowdale Road, Morgantown, WV, 26505, USA; 2 Johns Hopkins University Bloomberg School of Public Health, 615 N. Wolfe Street, Baltimore, MD 21205, USA; 3 Los Alamos National Laboratory, Los Alamos, NM 87545, USA

## Abstract

Accurate characterization of the physicochemical properties of aerosols generated for inhalation toxicology studies is essential for obtaining meaningful results. Great emphasis must also be placed on characterizing particle properties of materials as administered in inhalation studies. Thus, research is needed to identify a suite of techniques capable of characterizing the multiple particle properties (*i.e.*, size, mass, surface area, number) of a material that may influence toxicity. The purpose of this study was to characterize the morphology and investigate the size distribution of a model toxicant, beryllium. Beryllium metal, oxides, and alloy particles were aerodynamically size-separated using an aerosol cyclone, imaged dry using scanning electron microscopy (SEM), then characterized using phase contrast microscopy (PCM), a liquid suspension particle counter (LPC), and computer-controlled SEM (CCSEM). Beryllium metal powder was compact with smaller sub-micrometer size particles attached to the surface of larger particles, whereas the beryllium oxides and alloy particles were clusters of primary particles. As expected, the geometric mean (GM) diameter of metal powder determined using PCM decreased with aerodynamic size, but when suspended in liquid for LPC or CCSEM analysis, the GM diameter decreased by a factor of two (p < 0.001). This observation suggested that the smaller submicrometer size particles attached to the surface of larger particles and/or particle agglomerates detach in liquid, thereby shifting the particle size distribution downward. The GM diameters of the oxide materials were similar regardless of sizing technique, but observed differences were generally significant (p < 0.001). For oxides, aerodynamic cluster size will dictate deposition in the lung, but primary particle size may influence biological activity. The GM diameter of alloy particles determined using PCM became smaller with decreasing aerodynamic size fraction; however, when suspended in liquid for CCSEM and LPC analyses, GM particle size decreased by a factor of two (p < 0.001) suggesting that alloy particles detach in liquid. Detachment of particles in liquid could have significance for the expected versus actual size (and number) distribution of aerosol delivered to an exposure subject. Thus, a suite of complimentary analytical techniques may be necessary for estimating size distribution. Consideration should be given to thoroughly understanding the influence of any liquid vehicle which may alter the expected aerosol size distribution.

## Findings

Relating the physicochemical properties of an aerosol to its toxicity following inhalation is of specific interest for beryllium[1] and of general interest for a wide range of particles, including nanomaterials[2]. Various hypotheses have been put forth regarding particle mass, surface area, and number as metrics of toxicity of inhaled particles. Detailed control and physicochemical characterization of aerosols generated for inhalation toxicology studies is essential for obtaining meaningful study results.

A variety of techniques exist for exposing the lung to particles in pulmonary toxicology studies, including inhalation, intratracheal instillation, and pharyngeal aspiration[3]. A high research priority is the characterization of the exposure material as encountered by workers and as administered in a pulmonary toxicology study. Characterization of bulk material as produced or supplied may not accurately reflect the properties of the particles in the workplace atmosphere or of particles delivered to the lung by inhalation, instillation, or pharyngeal aspiration. For example, current methods for characterizing dry material using microscopy can reveal general morphology and particle size, but cannot determine whether material consists of solid particles or clusters, or whether material will deagglomerate in liquids such as lung surfactant. Similarly, current methods for characterizing material in suspension can reveal "equivalent" particle diameter, but cannot determine particle morphology. Thus, a suite of techniques is needed to understand the multiple particle properties that may influence toxicity.

The purpose of this study was to apply a suite of three different standard particle sizing techniques to investigate the size distributions of three physicochemical forms of beryllium and to assess the meaning and implication of any differences in the measured size distributions.

## Materials

We studied finished product metal powder (product type I-400, Brush Wellman Inc., Elmore, OH); finished product beryllium oxide (BeO) powder (product type UOX-125, Brush Wellman Inc.); process-sampled particles from the BeO powder production line; and process-sampled aerosol from an arc furnace in the copper-beryllium alloy production line. These materials were selected for study because they represent three industrially important chemical forms of beryllium and because inhalation of these materials is associated with development of chronic beryllium disease. The process-sampled BeO material is aerosol particles generated during screening of finished product BeO powder.

Bulk metal and BeO powders were aerosolized and aerodynamically size-separated using a 5-stage aerosol cyclone operated at 24 L min^-1 ^and 20°C; the 50% aerodynamic cutoff diameters (D_50_) were >6, 2.5, 1.7, 0.9, and 0.4 μm for stages 1 to 5 of the aerosol cyclone, respectively[4]. Process-sampled particles were collected from ventilation ductwork using a 5-stage aerosol cyclone operated at 28 L min^-1 ^and 23°C; D_50 _were >5.7, 2.3, 1.5, 0.7, 0.4 μm for stages 1 to 5 of the aerosol cyclone, respectively[1]. For the purposes of this study, we characterized material collected in stages 2, 3, and 4 of the aerosol cyclone. The density of the metal powders determined using pycnometry by Hoover et al. [4] were 1.89 g/cm^3 ^(stage 2), 1.89 g/cm^3 ^(stage 3), and 1.91 g/cm^3 ^(stage 4); the densities of the oxide powder and process-sampled BeO materials were not measured directly, but based on material purity [1], were taken to be the theoretical value for BeO of 3.0 g/cm^3^. The masses of processed sampled alloy particles collected were insufficient for direct measurement of density by pycnometry.

## Methods

Detailed morphology of the dry powders and particles was determined using scanning electron microscopy (SEM, Model 6300FXV, JEOL USA, Peabody, MA) operated at 5 kV. The SEM samples were prepared on 300 mesh gold electron microscopy grids coated with a lacey carbon substrate (Ted Pella Inc., Redding, CA).

The geometric mean (GM) particle size and geometric standard deviation (GSD) was determined using phase contrast microscopy (PCM), a liquid suspension particle counter (LPC), and computer-controlled scanning electron microscopy (CCSEM).

PCM (Carl Zeiss Inc., Germany) was used to determine particle Feret diameter (distance between tangents drawn from the extreme left and right edges of a particle measured perpendicular to a reference line) using a Filar ocular micrometer; the theoretical limit of resolution was 0.4 μm. Prior to analysis, the ocular micrometer was calibrated using a stage micrometer (10 μm scale division). For each material, 1000 particles were sized from slides of particles dispersed in mounting medium on a slide.

A calibrated LPC (Multisizer II, Coulter Electronics, Inc., Hialeah, FL) operated with 30 μm diameter aperture tube (0.6 – 18 μm actual cutoff range) was used to determine particle spherical equivalent diameter (spherical size equivalent to the volume of liquid displaced by a particle that produces the same voltage pulse when drawn between two electrodes). Prior to analysis, the LPC was calibrated using monodisperse 5-μm polystyrene latex spherical reference particles (Beckman-Coulter) and the calibration verified using monodisperse 2-μm polystyrene latex spherical reference particles (Beckman-Coulter). For analysis, a dilute suspension of each material in physiologic saline was subjected to ultrasonic agitation for 30 sec to mix, then an aliquot of each suspension was added to electrolyte solution (Coulter Electronics, Inc.); tens of thousands of particles were sized for each material. Size data were background corrected for particle counts in fluids. Additionally, an aliquot of suspension was deposited onto a 300 mesh gold electron microscopy grid (Ted Pella Inc.) and particles imaged using SEM (JEOL).

CCSEM (R.J. Lee Group, Monroeville, PA) analysis is expense relative to PCM and LPC techniques; therefore, we used CCSEM for analysis of material collected in stage 4 of the aerosol cyclone only to confirm that these small particles were not missed during sizing with PCM [5]. Using CCSEM, particle projected area diameter was determined with the rotated chord technique (length of 16 chords traced across the particle at equiangular intervals through the centroid of the particle) and ZepAPA computer software program algorithm (R.J. Lee Group). For analysis, a dilute suspension of each beryllium material in distilled and deionized water was deposited onto a polycarbonate filter using vacuum filtration and coated with a thin layer of gold/palladium; 1000 particles were sized from a representative section of each filter. Images of particles were obtained by depositing an aliquot of suspension onto a 300-mesh gold electron microscopy grid (Ted Pella Inc.) and analyzed using SEM (JEOL).

## Results and discussion

**Figure 1:**
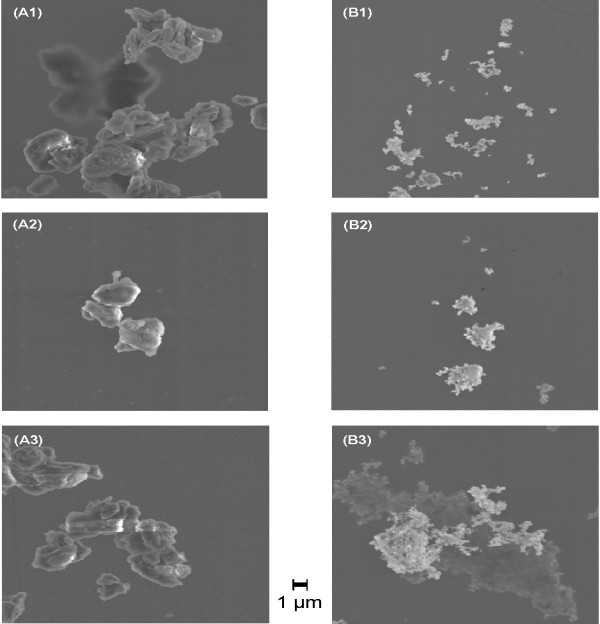
Representative SEM images of the different particle sizing preparations of beryllium metal and beryllium oxide evaluated in the study. (A1) shows the dry metal powder illustrating compact morphology with smaller sub-micrometer size particles attached to the surface of larger particles; (A2) metal powder suspended in liquid for sizing with a LPC illustrating detachment of smaller submicrometer size metal particles from the surface of larger particles and/or detachment of particle agglomerates; and (A3) metal powder suspended in liquid for sizing with CCSEM illustrating similar morphology as observed for the LPC suspension. Similarly, (B1) shows the dry oxide powder having cluster morphology; (B2) oxide powder suspended in liquid for LPC analysis illustrating similar morphology to dry oxide powder; and (B3) oxide powder suspended in liquid for CCSEM analysis illustrating similar morphology to dry oxide powder and powder for LPC analysis.

From SEM analysis, the dry metal powder was compact with smaller sub-micrometer size particles attached to the surface of larger particles (see Figure 1A1); BeO powder and process-sampled particles were clusters of primary particles (see Figure 1B1); alloy was also clusters of primary particles (data not shown).

Table 1 summarizes the GM (and GSD) particle size of beryllium materials by each sizing technique. As expected, the GM Feret diameter of metal powder became smaller with decreasing aerodynamic size-fraction; however, when suspended in liquid, the GM particle size often further decreased by half (p < 0.001). A small portion of this decrease can be attributed to differences in the definition of size among the three analytical techniques and/or our inability to size small particles using PCM. For example, Stein [5], in studies using polystyrene latex spheres observed that sizing using PCM at the theoretical limit of resolution of the objective lens (0.4 μm) missed 10% of particles relative to SEM. Additionally, particle physicochemical properties were not likely to contribute to the observed decrease in beryllium metal particle size between PCM and the wet sizing techniques. For example, the LPC measures the increase in electrical impedance that is proportional to the volume of liquid displaced by a particle, which is independent of particle color, refractive index, shape, or chemical composition. A more likely explanation for observed decrease in metal particle size is the sample preparation protocol, including the use of a liquid vehicle. Maier et al. [6] report that ultrasonic agitation of nanostructured titanium dioxide (TiO_2_) powder suspended in artificial lung fluid, dipalmitoyl phosphatidyl-choline (DPPC) dispersed in physiological saline, broke apart particles and formed an ultrafine particle fraction. Thus, it is possible that ultrasonic agitation of suspensions prior to LPC analysis might have broken apart particles (see Figure 1A2), resulting in the observed downward shift in the particle size distribution. Interestingly, for beryllium metal, particle size also shifted downward without the use of ultrasonic agitation prior to CCSEM analysis (see Figure 1A3). Thus, a plausible explanation for the LPC and CCSEM results is that smaller submicrometer size metal particles attached to the surface of larger particles detached from the larger particles and/or particle agglomerates detached when suspended in liquid, thereby shifting the particle size distribution downward. Detachment of metal particles could significantly change the expected aerosol size distribution when liquid is used as the vehicle for particle delivery to an exposure subject.

**Table 1:** Geometric mean diameter of beryllium powders and process-sampled particles

	Cyclone		Geometric mean, μm (GSD)^A^		
	
Material	Stage	D_50 _(μm)	PCM	CCSEM	LPC
Be metal powder	2	2.5	3.0 (1.8)	NP^B^	1.5 (1.8)
	3	1.7	2.7 (1.7)	NP	1.3 (1.5)
	4	0.9	2.0 (1.5)	1.1 (1.9)	1.1 (1.6)
BeO powder	2	2.5	1.0 (1.3)	NP	1.1 (1.3)
	3	1.7	1.2 (1.5)	NP	1.0 (1.3)
	4	0.9	1.0 (1.4)	0.9 (1.5)	1.0 (1.3)
Process BeO	2	2.3	1.2 (1.6)	NP	--^C^
	3	1.5	1.1 (1.6)	NP	--
	4	0.7	1.2 (1.6)	1.0 (1.9)	--
Process alloy	2	2.3	2.1 (1.9)	NP	1.1 (1.3)
	3	1.5	1.9 (1.7)	NP	1.0 (1.3)
	4	0.7	1.6 (1.7)	--^D^	0.9 (1.3)

^A ^GSD = geometric standard deviation

^B ^NP = analysis not performed for this size-fraction

^C ^For the sake of efficiency in experimental design, separate LPC analyses were not performed for the process BeO material because the BeO powder material was considered representative of the process BeO material.

^D ^Greater than 60% of these particles were 0.4–0.7 μm precluding graphical determination of the GM

The size of each BeO material appeared similar among sizing techniques (see Figures 1, images B1 to B3); however, values determined using PCM and the LPC were statistically different (p < 0.001), with the exception of stage 4 BeO powder (p = 0.49). For BeO, these small differences in GM particle size may be attributed to differences in particle size definition. For these materials, aerodynamic cluster size will dictate deposition in the lung, but primary particle size may influence biological activity.

The GM Feret diameter of particles sampled from the alloy production line became slightly smaller with decreasing aerodynamic size fraction; however, when suspended in liquid for CCSEM and LPC analyses, GM particle size decreased by up to a factor of two (p < 0.001). Thus, these beryllium-containing alloy particles probably also detach in liquid, thereby shifting the size distribution smaller.

The detachment of particles suspended in a liquid vehicle has significance for the expected versus actual size (and concomitant number) distribution of particles delivered to an exposure subject. In the current study, beryllium metal and beryllium alloy particles, but not beryllium oxide, detached in deionized water (in the absence of external agitation) and in physiological saline mixed with an electrolyte solution (after brief ultrasonic agitation). Maier et al. [6] report that in the absence of ultrasonic agitation, nanostructured TiO_2 _powder suspended in artificial lung fluid did not disaggregate, but when subjected to brief ultrasonic agitation an ultrafine particle size fraction formed. Sager et al. [7] report TiO_2 _nanoparticles and ultrafine carbon black exhibit varying degrees of dispersability in liquids such as physiologic saline, artificial lung fluid, and rat and mouse bronchoalveolar lavage (BAL) fluid. Thus, on a material-by-material basis, detailed knowledge of the effect of the sample preparation conditions, including the use of a liquid vehicle (water, physiological saline, artificial lung surfactant, BAL fluid, etc.), on measured physicochemical characteristics of aerosols generated for toxicology studies is essential.

We conclude that careful attention is needed when characterizing exposure aerosols for pulmonary toxicology studies. A suite of complimentary analytical techniques may be necessary for understanding particle size distribution. When estimating size distribution, consideration must be given to the use of a liquid vehicle, which may alter the expected aerosol size distribution.

## Competing interests

The author(s) declare that they have no competing interests.

## Authors' contributions

ABS conceived, designed, acquired, analyzed, interpreted the data, and drafted the manuscript. MDH aerodynamically size-separated the powders, interpreted the data, and drafted the manuscript. RMD imaged the study materials and interpreted the data. GAD collected the process-sampled particles. PNB drafted the manuscript. RCS collected the process-sampled particles and drafted the manuscript. All authors read and approved the final manuscript.
